# Mediator Subunit Med28 Is Essential for Mouse Peri-Implantation Development and Pluripotency

**DOI:** 10.1371/journal.pone.0140192

**Published:** 2015-10-07

**Authors:** Lin Li, Ryan M. Walsh, Vilas Wagh, Marianne F. James, Roberta L. Beauchamp, Yuh-Shin Chang, James F. Gusella, Konrad Hochedlinger, Vijaya Ramesh

**Affiliations:** 1 Center for Human Genetic Research, Massachusetts General Hospital, Boston, Massachusetts, United States of America; 2 Center for Regenerative Medicine, Massachusetts General Hospital, Boston, Massachusetts, United States of America; 3 Cardiovascular Research Center, Massachusetts General Hospital, Boston, Massachusetts, United States of America; 4 Harvard Stem Cell Institute, Cambridge, Massachusetts, United States of America; University of Kansas Medical Center, UNITED STATES

## Abstract

The multi-subunit mammalian Mediator complex acts as an integrator of transcriptional regulation by RNA Polymerase II, and has emerged as a master coordinator of development and cell fate determination. We previously identified the Mediator subunit, MED28, as a cytosolic binding partner of merlin, the Neurofibromatosis 2 (NF2) tumor suppressor, and thus MED28 is distinct in having a cytosolic role as an NF2 interacting protein as well as a nuclear role as a Mediator complex subunit. Although limited *in vitro* studies have been performed on MED28, its *in vivo* function remains unknown. Employing a knockout mouse model, we describe for the first time the requirement for Med28 in the developing mouse embryo. Med28-deficiency causes peri-implantation lethality resulting from the loss of pluripotency of the inner cell mass accompanied by reduced expression of key pluripotency transcription factors *Oct4* and *Nanog*. Further, overexpression of Med28 in mouse embryonic fibroblasts enhances the efficiency of their reprogramming to pluripotency. Cre-mediated inactivation of *Med28* in induced pluripotent stem cells shows that Med28 is required for their survival. Intriguingly, heterozygous loss of *Med28* results in differentiation of induced pluripotent stem cells into extraembryonic trophectoderm and primitive endoderm lineages. Our findings document the essential role of Med28 in the developing embryo as well as in acquisition and maintenance of pluripotency during reprogramming.

## Introduction

Mediator is a large evolutionarily conserved protein complex comprising ~30 distinct subunits that plays a pivotal role in the regulation of eukaryotic mRNA synthesis. Although initially identified by its ability to support activator-dependent transcription, Mediator is part of the core transcriptional machinery and plays important roles at nearly all stages of transcription, from recruiting RNA polymerase II (Pol II) to promoters and initiating transcription to assisting efficient elongation and processing of transcripts to produce mature mRNAs [1, 2]. Mediator subunit, MED28 (also named magicin) is an ~24 kDa protein expressed in many cell lines and tissues, which we identified previously as a binding partner of merlin, the Neurofibromatosis 2 (NF2) tumor suppressor [3]. Our earlier work also showed a role for MED28 in receptor-mediated signaling at the cell surface and an association with Src-family kinases such as Fyn, Src and Lck [4]. MED28 was independently identified as a differentially expressed gene in endothelial cells and named endothelial-derived gene 1 (EG–1), with elevated expression in cancerous epithelial cells including carcinomas of the breast, colon and prostate [5]. Elevated MED28 expression was associated with poor outcome in breast cancer patients, and targeted downregulation of MED28, either by siRNA or antibody, caused decreased breast cancer cell proliferation and reduced xenograft growth [6, 7].

Our earlier study also demonstrated that RNAi-mediated suppression of Med28 resulted in a significant induction of many genes involved in smooth muscle cell (SMC) differentiation, and Med28 suppression in the multipotent, mesenchymal-derived murine precursor cell line C2C12 caused transdifferentiation into SMCs [8]. A recent study employing a shRNA library to screen for regulators of transcription and chromatin necessary for maintaining murine embryonic stem (ES) cells identified many Mediator subunits including Med28 [9]. Mediator subunits Med1 and Med12 in complex with cohesin were shown to physically and functionally connect the enhancers and core promoters of active genes in murine ES cells [9].

MED28 is unique among Mediator subunits as one of the few subunits specific to higher eukaryotes and is localized to both the cytoskeletal fraction and the nucleus where it is presumably involved in gene transcription regulation as part of the Mediator complex. Thus MED28 may serve as a multi-faceted adaptor/scaffolding protein to relay cellular signals to the cytoskeleton, and from the cytoskeleton to the nucleus. To investigate the functions of Med28 *in vivo* and its potential role during mammalian development, we generated a mouse model carrying a conditional allele for *Med28 (Med28* ^*fl/fl*^). Constitutional deletion of *Med28* using a protamine-Cre transgenic line revealed that absence of Med28 leads to peri-implantation lethality. Homozygous *Med28*-deficient embryos develop to the blastocyst stage, but the inner cell mass (ICM) is not pluripotent. In the absence of Med28, mouse embryonic fibroblasts (MEFs) lose the capability to reprogram, and, consistent with this, Med28 overexpression augments reprogramming efficiency. Further, Cre-mediated removal of Med28 from induced pluripotent stem cells (iPSCs) shows that Med28 is required for their survival and intriguingly, heterozygous loss of Med28 causes aberrant differentiation of iPSCs.

## Results

### Generation of Med28 knockout mice

**Fig 1:**
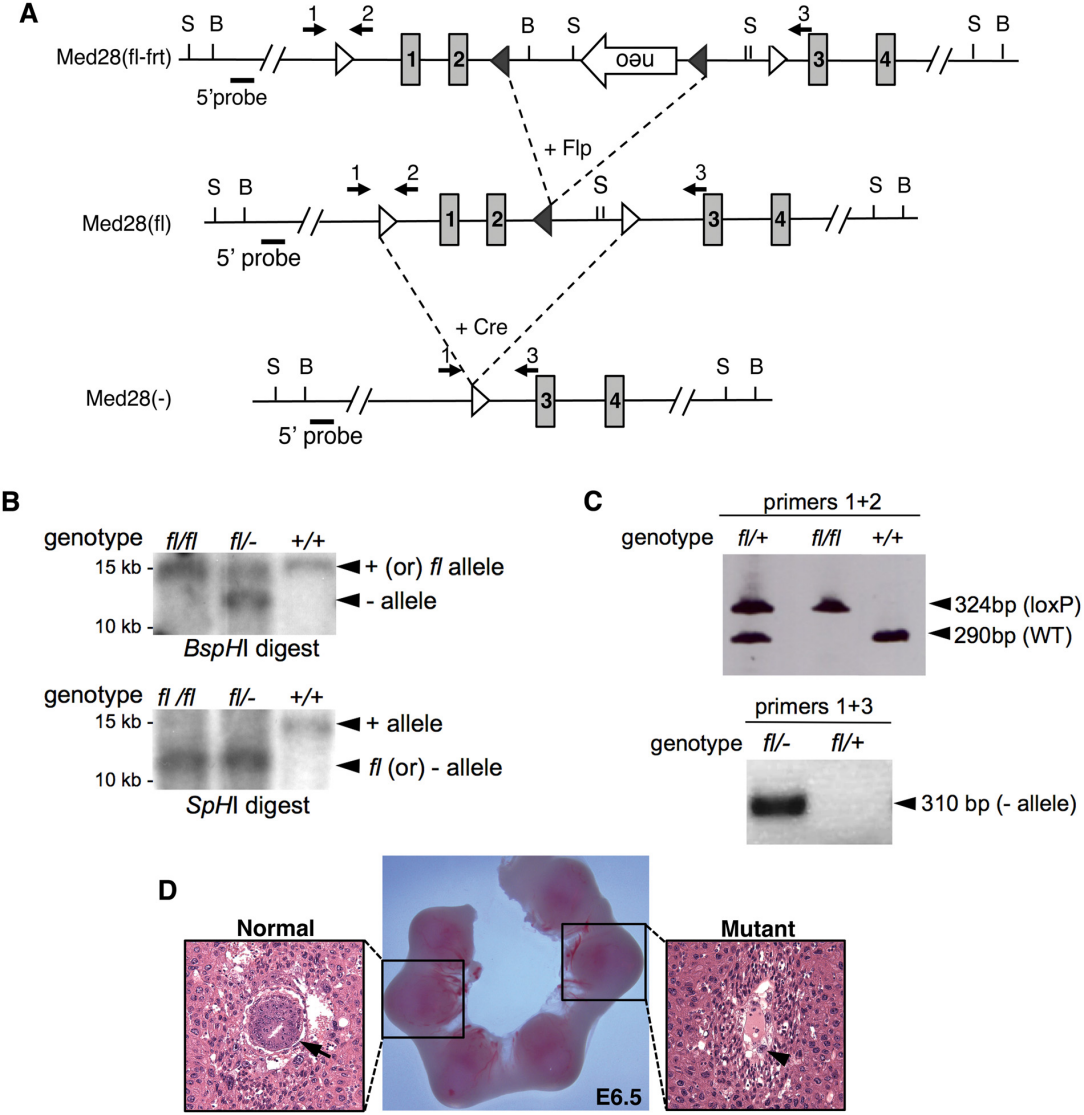
Targeted disruption of *Med28*. **(A)** Schematic representation of the targeted allele and null allele. Targeted *Med28* allele in mouse ES cells with neo cassette in the reverse orientation (top, Med28(fl-frt), targeted *Med28* allele without neo cassette after transient transfection with Flp (middle, Med28(fl)) and Med28 null allele with Exon 1 and 2 deletions after breeding with Protamine-Cre mouse (bottom, Med28(-)). Shaded boxes with numbers represent the exons. Open triangle: loxP site; closed triangle: Frt site; B: BspH1 restriction site; S: SpHI restriction site. Numbered arrows represent PCR primers used for genotyping. Short solid bar represents 5’ probe outside the homologous region used for Southern blot analysis. **(B)** Representative Southern blot analysis used to confirm correct targeting. DNA was digested with either BspHI or SpH1 restrict enzymes and the 5’Probe (shown in **A**) outside the homologous region was used to detect wildtype (+), floxed (fl) and null (-) alleles at expected sizes for WT (+) allele, 14.6 kb (BspHI) or 15.2 kb (SpHI); floxed allele without neo (fl), 15 kb (BspHI) or 12 kb (SpHI); and null (-) allele, 12.1 kb (BspHI) or 12.7 kb (SpHI). **(C)** Representative PCR analysis using primers 1 and 2 (shown in A) confirm the presence of 5’loxP site (top panel, 324 bp) and WT allele (top panel, 290 bp). PCR analysis using primers 1 and 3 (shown in **A**) confirm presence of only the null (-) allele (bottom panel, 310 bp). **(D)** Histological analysis of 6.5-dpc (E6.5) presumed to be Med28 mutant embryos shows disorganized extraembryonic tissue with no discernible epiblast. Sections from whole decidual swellings were stained with hematoxylin and eosin. Arrow, epiblast; arrowhead, trophoblast giant cell.

*Med28* spans 4 exons encoding a protein of 178 amino acids with the translation start site in exon 1 and a 3’ UTR spanning most of exon 4. Med28 is widely expressed during murine embryonic development in various tissues including the CNS, spinal cord, dorsal root ganglion, muscle precursors in the limbs, somites, and heart (S1 Fig). To study the *in vivo* function of Med28 during mammalian development, we generated a targeting vector with two loxP sites flanking *Med28* exons 1 and 2, as well as a neomycin (Neo) resistance cassette in reverse orientation within intron 2, flanked by Frt sites (Fig 1A). The targeting vector was introduced into 129/JV ES cells by electroporation, and drug-resistant colonies were screened for homologous recombination. Of 198 colonies screened by PCR, we identified 3 correctly targeted clones that were confirmed by Southern blots (data not shown). The existence of a Neo cassette can produce a hypomorphic allele even in the reverse orientation; therefore, to remove the Neo cassette, one clone with normal karyotype was chosen for transfection with Flp recombinase. This was followed by culturing of 98 transfected clones in duplicate, with and without G418. Based on G418 selection, we chose 4 clones for genotyping by PCR and Southern blot analyses, and confirmed correct targeting, i.e. *Med28* ^*fl/+*^ ES cells with loxP sites flanking exons 1 and 2 lacking the Neo cassette (Fig 1). Two clones were chosen for injection into C57BL/6 blastocysts, which yielded successful germline transmission. We then generated *Med28* conditional allele mice (*Med28* ^*fl/fl*^ *)* by intercrossing *Med28* ^*fll+*^ mice, and confirmed their genotype by Southern blot and PCR analyses (Fig 1B and 1C). Heterozygous *Med28* ^*fl/+*^ mice were mated with protamine-Cre mice to generate double heterozygous *Med28* ^*fl/+*, *Cre/+*^ mice, selectively deleting *Med28* in male germ cells [10]. These mice were then crossed with either *Med28* ^*fl/fl*^ or B6 mice to generate *Med28* ^*fl/-*^ or *Med28* ^*+/-*^ mice. Both heterozygous mutant mice (*Med28* ^*fl/-*^ or *Med28* ^*+/-*^) were viable, fertile and phenotypically normal, with no observable differences between the two lines.

### Med28-deficiency causes peri-implantation lethality associated with defective inner cell mass (ICM) proliferation

Genotyping of progeny from intercrossed *Med28* heterozygotes (*Med28* ^*fl/-*^ or *Med28* ^*+/-*^ *)* demonstrated that among 271 pups born, 166 (61%) were heterozygous for a null allele and 105 (39%) were wildtype (*Med28* ^*fl/fl*^ or *Med28* ^*+/+*^). The complete absence of homozygous knockout (*Med28* ^*−/−*^ *)* mice is a statistically significant deviation from the expected ratio (p<0.0001), suggesting that a homozygous *Med28*-null genotype is embryonic lethal. Crossing *Med28* heterozygotes with wildtype mice, produced 63 offspring of which 34 (54%) were wildtype and 29 (46%) were heterozygotes, consistent with the expected ratio of 1:1 (p = 0.53), and indicating that heterozygotes do not have reduced viability. Harvesting embryos at various developmental stages revealed that mutant embryos fail to survive shortly after implantation (Table 1). Hematoxylin-eosin-stained sections of whole decidual swellings at ~6.5 days post-coitus (dpc) from *Med28* heterozygote intercrosses showed that all blastocysts appeared to correctly implant. However, other than disorganized extraembryonic tissues with evident trophoblast giant cells, mutant embryos showed no egg cylinder structure and lacked an epiblast (Fig 1D). These data indicate that *Med28* deficiency causes peri-implantation lethality, supporting a crucial role for Med28 during early embryogenesis.

**Table 1:** Genotyping analysis of offspring and embryos derived from *Med28* heterozygote intercrosses.

	Total	WT (1/4)	Het (1/2)	KO (1/4)	Unknown^a^
**Pups**	271	105	166	0	0
**E12.5**	18	5	10	0	3
**E8.5**	18	5	8	0	5
**E7.5**	13	4	9	0	0
**E6.5**	9	2	4	0	3
**E3.5**	53	14	23	12	4

^a^Empty deciduas or not enough DNA to genotype

**Fig 2:**
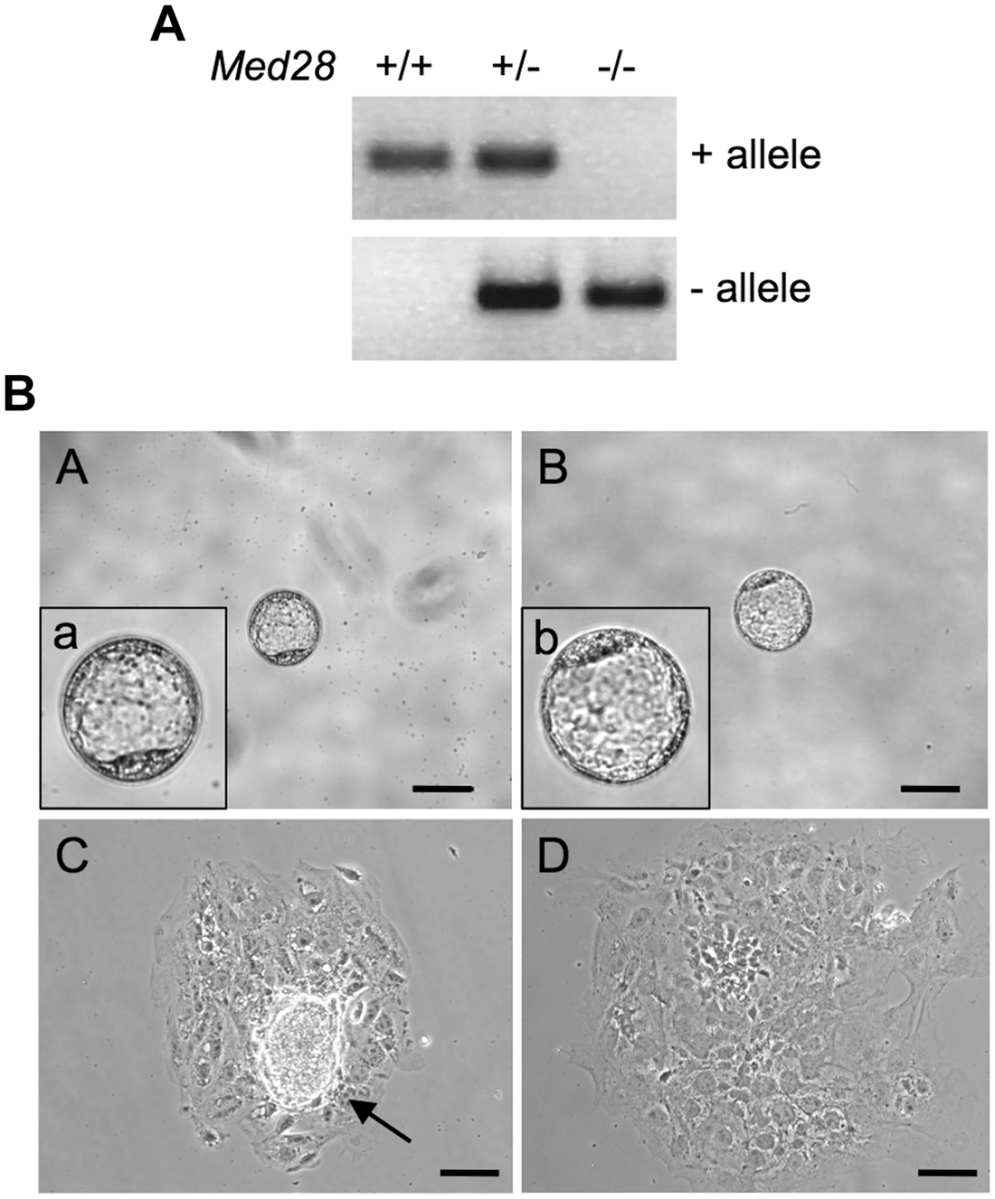
Impaired ICM outgrowth of *Med28* ^*-/-*^ blastocysts. **(A)** Representative PCR genotyping of E3.5 blastocysts from *Med28* ^*+/-*^ intercrosses show WT (+) allele and null (-) allele. Primers described in Fig 1C are used for genotyping. **(B)** Phase contrast microscopy images of embryos from *Med28* ^*+/-*^ intercrosses. At embryonic day E3.5, *Med28* ^*+/+*^ blastocysts (panel A; a, enlarged inset) are indistinguishable from *Med28* ^*-/-*^ blastocysts (panel B; b, enlarged inset). After 3 days in culture (DIV3), unlike *Med28* ^*+/+*^ blastocysts that show outgrowth (panel C, arrow), ICMs from *Med28* ^*-/-*^ blastocysts fail to show outgrowth (panel D). A total of 14 WT and 12 mutant blastocysts were examined. Scale bar, 100 μm.

Because of the peri-implantation lethality, we isolated and cultured 3.5 dpc (E3.5) blastocysts from *Med28* heterozygote intercrosses. PCR genotyping revealed that of the 53 blastocysts examined, 12 (22.6%) were homozygous *Med28* knockout and did not significantly differ from the expected Mendelian ratio of 1:3 (p = 0.84) (Table 1). A representative genotype is shown in Fig 2A. *Med28*-null blastocysts were initially indistinguishable from WT and heterozygous embryos (Fig 2B). However, after culturing 49 blastocysts in standard ES cell medium with 15% FBS and LIF for 3 days *in vitro* (DIV3), all 14 wildtype and 23 heterozygous blastocysts exhibited a distinct inner cell mass (ICM) that increased in size during the culture period (Fig 2B). In contrast, none of the 12 *Med28-*null cultured blastocysts examined showed well-developed ICM outgrowths (Fig 2B). This phenotype is strikingly similar to embryos that lack pluripotency factors Oct4, Nanog and Sox2 as well as FGF4 [11–14], suggesting that Med28 is essential for maintenance and proliferation of the ICM.

### ICMs from *Med28* ^*-/-*^ are not pluripotent

**Fig 3:**
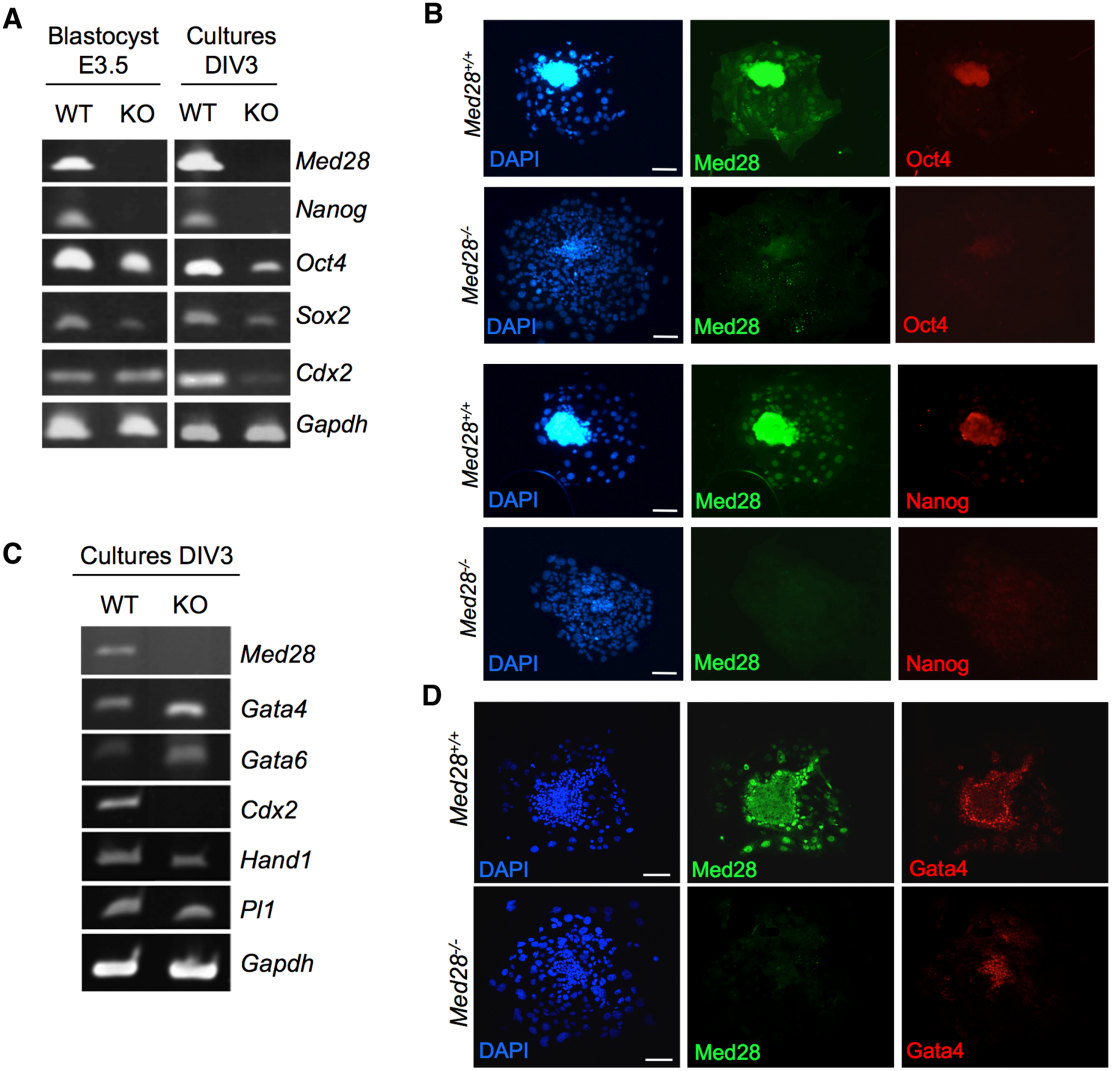
*Med28* ^*-/-*^ ICM is not pluripotent. **(A)** Semi-quantitative RT-PCR demonstrates reduced expression of pluripotency markers Oct4, Nanog and Sox2 in *Med28* ^*-/-*^ (KO) E3.5 blastocysts and cultured blastocysts DIV3 compared to wildtype (WT) controls. Trophoectoderm marker *Cdx2* expression is reduced in cultured *Med28* ^*-/-*^ (KO) blastocysts. **(B)** Immunofluorescence analysis shows reduced expression of Oct4 (red, top panels) and Nanog (red, bottom panels) in cultured *Med28* ^*-/-*^ blastocysts compared to wildtype controls. **(C)** Semi-quantitative RT-PCR demonstrates increased expression of primitive endoderm markers *Gata4* and *Gata6* in DIV3 cultured *Med28* ^*-/-*^ (KO) blastocysts, while trophoblast giant cell markers *Hand1* and *Pl1* expressions are not up-regulated compared to WT control. *Gapdh* expression serves as a control (shown in **A** and **C**). **(D)** Confocal microscopy images of immunofluorescence analysis show that Gata4 is expressed in primitive endoderm cells surrounding the ICM in DIV3 cultured *Med28* ^*+/+*^ blastocysts (red, top panel) and *Med28* ^*-/-*^ blastocysts (red, bottom panel). Nuclear DAPI (blue) and Med28 (green) are also shown (shown in **B** and **D**). Scale bar, 100 μm. At least three independent experiments were carried out for all data sets.

To examine whether pluripotency was affected in *Med28* ^*-/-*^ ICMs, we performed semi-quantitative RT-PCR analyses on at least 3 WT and 3 mutants. Blastocysts, both intact (E3.5) and cultured (DIV3), showed reduced expression of pluripotency markers *Oct4*, *Nanog and Sox2* indicating that ICMs from *Med28* ^*-/-*^ lack pluripotency (Fig 3A). Immunofluorescence (IF) analysis of cultured blastocysts further confirmed reduced expression of pluripotency markers Oct4 and Nanog (Fig 3B), suggesting that *Med28* ^*-/-*^ ICMs are unable to maintain an undifferentiated state when cultured *in vitro*. Similar to Oct4, Nanog and Sox2 [11–13], Med28 appears to be essential for the establishment of pluripotency in the ICM.

During normal blastocyst development, very early internal/apolar cells go on to form the ICM, which further segregates into the epiblast (EPI) or embryonic lineage, and the primitive endoderm (PE), whereas the outer/polar cells form the trophoectoderm (TE) give rise to the trophoblast that contributes to extraembryonic tissues, such as fetal placenta [15]. Oct4-deficient internal cells differentiate into trophoblast giant cells while the Nanog-deficient ICM differentiate into PE-like cells [12, 13, 16]. *Med28*-deficient embryos demonstrated reduced expression of both *Oct4* and *Nanog*; we therefore examined various early lineage markers in *Med28* ^*-/-*^ intact (E3.5) and cultured (DIV3) blastocysts. The TE-specific transcription factor *Cdx2* was unchanged in *Med28* ^*-/-*^ blastocysts (E3.5) suggesting that the defect caused by *Med28-*deficiency is specific to the ICM (Fig 3A, left panel). However, upon culturing, *Cdx2* expression was decreased in *Med28*-null DIV3 blastocysts indicating that normal trophoblast proliferation may not be maintained (Fig 3A, right panel and Fig 3C). Furthermore, compared to wildtype, markers of differentiated trophoblast giant cells *Hand1* and *Pl–1* [17] were not upregulated in *Med28*-null DIV3 blastocysts suggesting that, unlike *Oct4*-deficiency, *Med28*-deficient cells may not differentiate into trophoblast giant cells (Fig 3C). In contrast, similar to *Nanog*-deficiency, increased expression of PE transcription factors *Gata4* and *Gata6* was observed in *Med28*-null compared to wildtype DIV3 blastocysts, suggesting that *Med28-*null ICM may be prone to differentiate into PE cells (Fig 3C). IF analysis in cultured blastocysts showed that Gata4 is expressed in primitive endoderm cells surrounding ICM in wildtype embryos as previously described [18]. Gata4 expression was maintained in *Med28*-null embryos (Fig 3D). Our results are consistent with lack of epiblast as a source of FGF4 for blastocyst outgrowth/trophoblast proliferation, and the presence of GATA4 expressing cells [19]. Taken together, these findings confirm that Med28 is essential for early embryonic development through regulating the establishment of pluripotency in the ICM.

### Med28-deleted MEFs fail to reprogram

**Fig 4:**
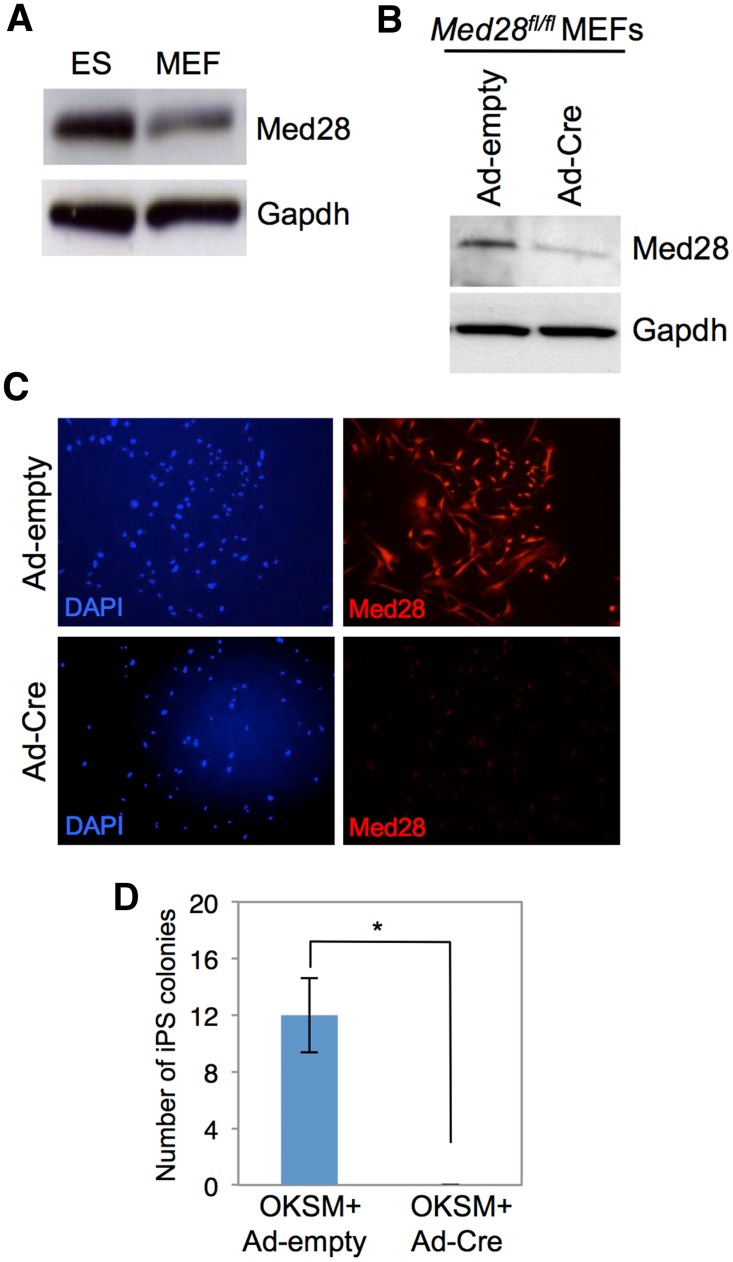
Loss of Med28 in MEFs results in lack of reprogramming. **(A)** Western blot analysis shows that Med28 expression is much higher in ES cells compared to MEFs. **(B)** Western blot shows reduced Med28 expression in *Med28* ^*fl/fl*^ MEFs at 6 days post-infection with Ad-Cre compared to Ad-empty control adenovirus. **(C)** Immunofluorescent staining of *Med28* ^*fl/fl*^ MEFs demonstrates significant reduction of Med28 (red) in Ad-Cre-infected cells compared to Ad-empty control. DAPI staining (blue) of cell nuclei is shown. **(D)** Quantitation of number of iPSC colonies (triplicate experiments) from *Med28* ^*fl/fl*^ primary MEFs infected with OKSM and rtTA followed by infection with Ad-empty (OKSM+Ad-empty) or Ad-Cre (OKSM+Ad-Cre) one day after Dox treatment. Note that infection with OKSM+Ad-Cre produced no colonies. Data are presented as mean +/- SD (*p<0.05).

Med28 is expressed in MEFs, but at a lower level than in ES cells, similar to findings of Med1 [9] (Fig 4A). To further understand Med28 function, we derived primary MEFs from E13.5 embryos of *Med28* ^*fl/fl*^ intercrosses. *Med28* was removed in the MEFs by infecting with an adenovirus vector expressing the Cre transgene (Ad-Cre). Ad-Cre infected MEFs displayed a significant reduction in Med28 expression (Fig 4B and 4C). *Med28* ^*fl/fl*^ primary MEFs were infected using the “stem cell cassette” (STEMCCA), a single lentiviral vector that expresses doxycycline (Dox)-inducible Oct4, Klf4, Sox2 and cMyc (OKSM factors) [20] and rtTA (reverse tetracycline-dependent transactivator), a co-activator of the Dox inducible system. Transduced *Med28* ^*fl/fl*^ MEFs were then treated with Dox for 12 days followed by removal of Dox and culturing for an additional 5 days. *Med28* ^*fl/fl*^ MEFs were reprogrammable as indicated by alkaline phosphatase (AP)-positive iPSC colonies (data not shown). To test the effects of *Med28* loss on reprogramming, we carried out *Med28* ^*fl/fl*^ MEF reprogramming as above with additional infection of Ad-Cre or Ad-empty control virus. Deletion of Med28 in OKSM-expressing MEFs (Ad-Cre) did not show significant change in cell number when compared to control (Ad-empty) MEFs, suggesting no difference in proliferation rate (S2A Fig); however, *Med28*-deleted (Ad-Cre) MEFs were unable to generate iPSC colonies (Fig 4D).

### Med28 overexpression facilitates generation of iPSC

**Fig 5:**
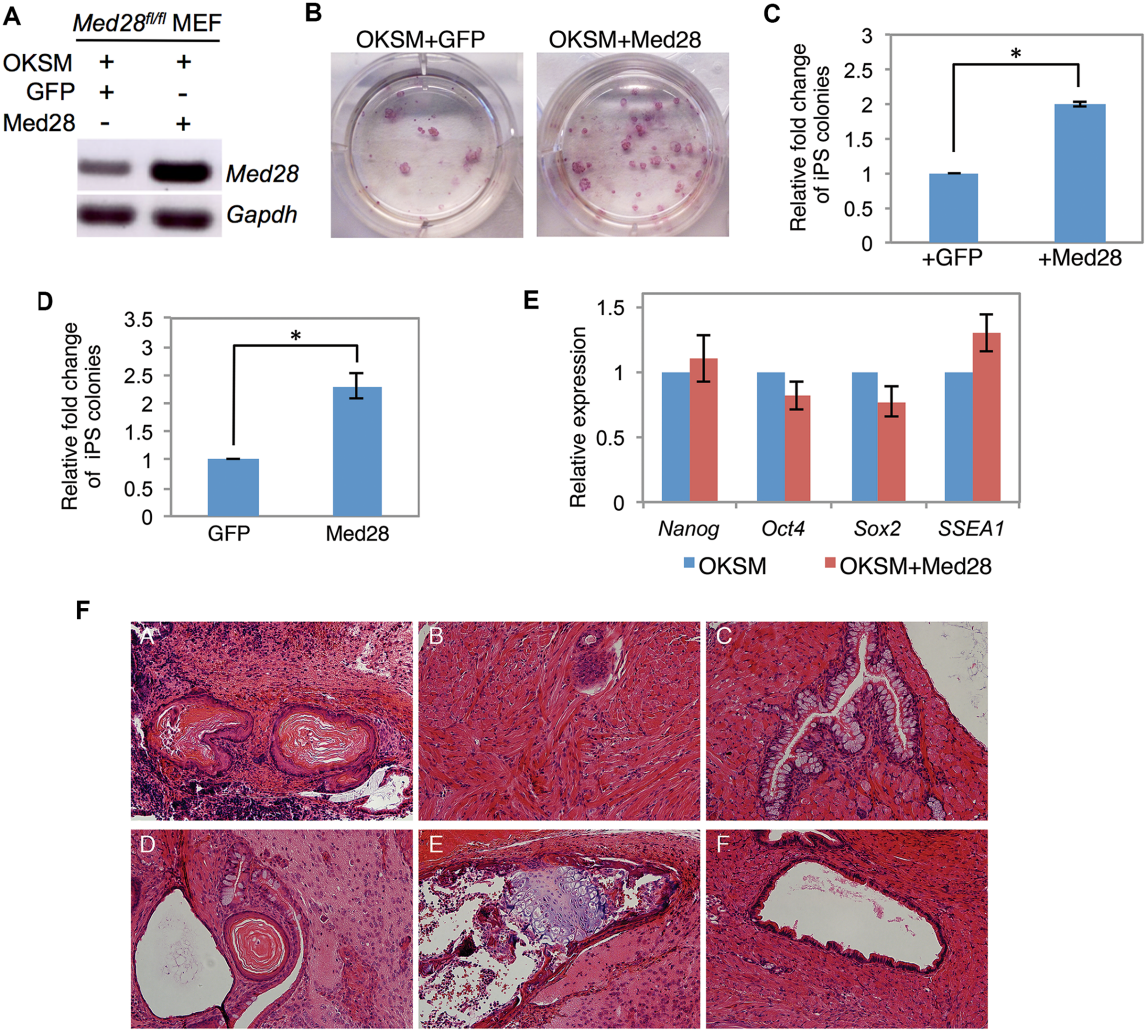
Med28 overexpression enhances reprogramming. **(A)** RT-PCR of *Med28* ^*fl/fl*^ MEFs shows increased *Med28* expression 4 days post infection of OKSM and Med28 compared to OKSM and GFP. *Gapdh* serves as a control. **(B)** Representative images from triplicate experiments show more AP-positive iPSC colonies upon Med28 overexpression (OKSM+Med28) compared to control (OKSM+GFP). **(C)** Relative fold change of AP-positive iPS colonies shows ~ 2-fold increase upon co-infection of OKSM, rtTA and Med28 (+Med28) compared to co-infection of OKSM, rtTA and GFP (+GFP). **(D)** Relative fold change of AP-positive iPS colonies from OKSM-MEF (derived from transgenic mouse line with integrated OKSM and rtTA) infected with either GFP or Med28 shows about a two-fold increase with Med28 overexpression. **(E)** Real-time PCR from single iPS colonies picked from *Med28* ^*fl/fl*^ MEFs infected with OKSM (n = 3) or OKSM+Med28 (n = 3). Pluripotency markers *Nanog*, *Oct4*, *Sox2* and *SSEA1* expression are comparable in control (OKSM) and OKSM+Med28 iPSC colonies. **(F)** Hematoxylin and eosin staining of teratomas derived from OKSM control (panels A-C) and OKSM+Med28 iPSC (panels D-F). iPSCs revealed differentiation into all three germ layers including ectoderm (panels A and D), mesoderm (panels B and E) and endoderm (panels C and F). Data are presented as mean +/- SD (shown in **C**, **D** and **E**) (*p<0.05).

We next examined the effect of *Med28* overexpression on reprogramming. *Med28* or *GFP* (as a control) were cloned into the same Dox-inducible vector backbone as STEMCCA and co-infected with STEMCCA and rtTA into *Med28* ^fl/fl^ MEFs (Fig 5A). We observed a ~2-fold increase in reprogramming efficiency upon *Med28* overexpression compared to GFP control (Fig 5B and 5C). After 5 days Dox treatment, the number of Med28-overexpressing MEFs was not significantly greater than control MEFs, suggesting that the increased reprogramming efficiency was not simply due to increased proliferation (S2B Fig). To confirm the effect of *Med28* overexpression on reprogramming, we also overexpressed *Med28* in a MEF cell line derived from a transgenic mouse with integrated rtTA and the OKSM cassette [21], and observed a similar two-fold increase in reprogramming efficiency (Fig 5D).

Single iPSC colonies from three independent clones derived from *Med28-*overexpressing MEFs (OKSM+Med28) were cultured, and semi-quantitative RT-PCR analysis showed expression of pluripotency markers *Nanog*, *Oct4*, *Sox2*, and *SSEA1* that was comparable to OKSM expression alone (Fig 5E). In addition, subcutaneous injection of *Med28-*overexpressing iPSCs into flanks of SCID mice generated teratomas containing all three germ layers determined by histological analyses (Fig 5F). These experiments indicate that the iPSCs derived from *Med28-*overexpressing MEFs are pluripotent both *in vitro and in vivo*. To test whether the rate of iPSC reprogramming was increased, we subjected *Med28*-overexpressing MEFs to a shortened Dox treatment (6 days) but no increase in iPSC colony formation was observed compared to STEMCCA alone. This suggests that *Med28* overexpression increases the efficiency of reprogramming, but not its kinetics (data not shown) and further supports the essential role of Med28 in pluripotency.

### Dosage sensitive role of Med28 in iPSCs

**Fig 6:**
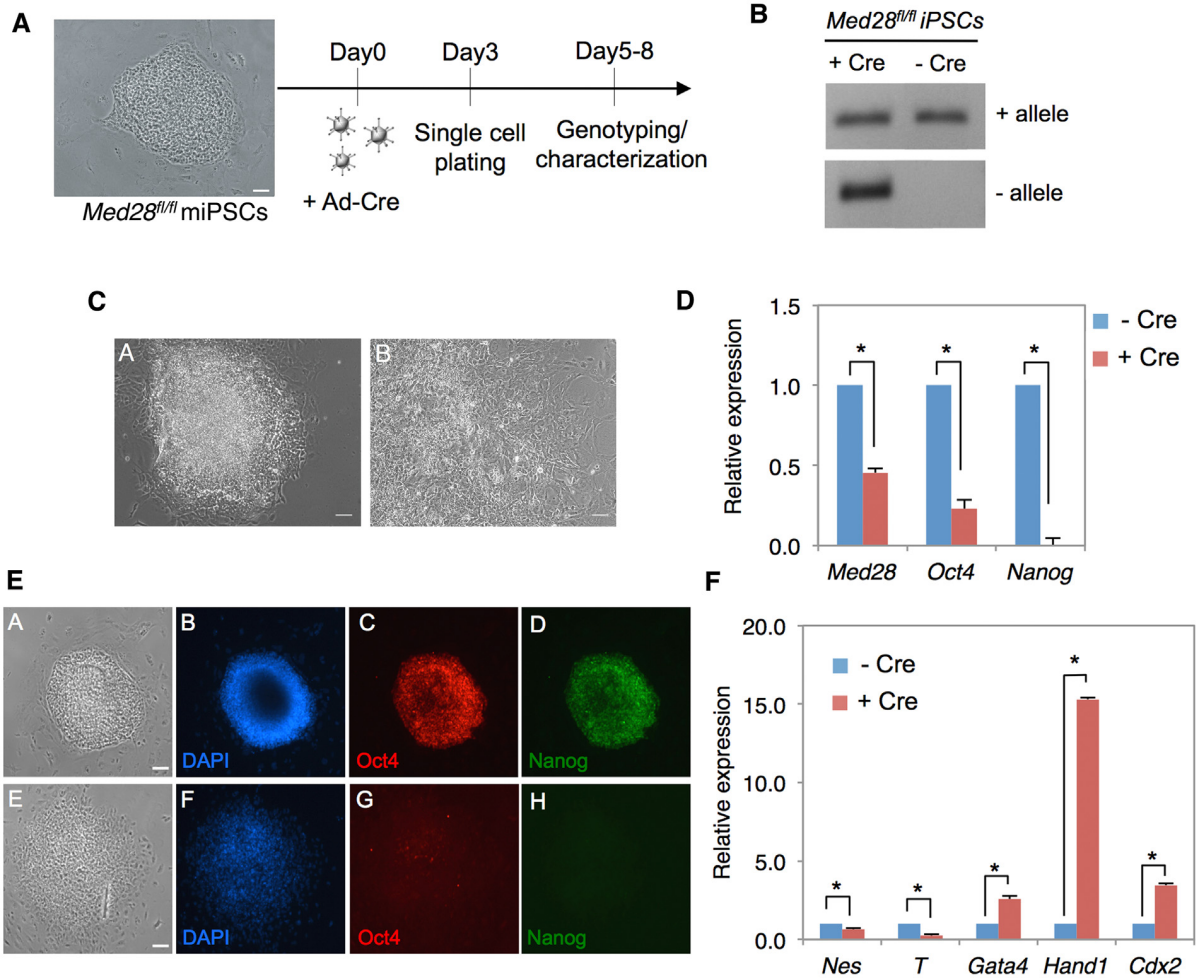
Cre-mediated heterozygous deletion of Med28 causes differentiation of iPSC. **(A)** *Med28* ^*fl/fl*^ iPSCs were infected with Ad-Cre, followed by single cell plating on day 3 and genotyping along with other analyses performed between days 5–8. **(B)** PCR genotyping shows clones with both deleted (- allele) and non-deleted WT (+ allele) alleles infected with Ad-Cre (+ Cre) compared to uninfected clones (-Cre), respectively. **(C)** Representative bright field images show noticeable differences in morphology between non-deleted (panel A, -Cre) and deleted (panel B, +Cre) *Med28* ^*fl/fl*^ iPSC colonies (scale bar 200μm). Note the differentiated colony morphology of the *Med28* ^*fl/fl*^ (panel B, +Cre) cells. **(D)** Quantitation of real-time PCR for deleted *Med28* ^*fl/fl*^ iPSC colonies (+Cre) show ~2-fold decreased expression of *Med28* as well as significantly lower expression of *Oct4* and *Nanog* (n = 4; *, p<0.05) compared to non-deleted *Med28* ^*fl/fl*^ iPSC (-Cre) colonies. **(E)** Immunostaining of deleted *Med28* ^*fl/fl*^ colonies (panels E-H) show loss of self-renewal factor Oct4 (red, C and G) and Nanog (green, D and H) compared to non-deleted colonies (panels A-D). Bright field images (A and E) and nuclear DAPI (blue, B and F) are also shown (scale bar 200μm). **(F)** Quantitation of real—time PCR for differentiation markers reveal increased differentiation markers in deleted (+Cre) *Med28* ^*fl/fl*^ iPSC colonies for extra embryonic lineage (*Gata4*, *Hand1*, and *Cdx2*) and decreased germ layer lineage makers nestin (Nes, ectoderm) and T brachyury (T, mesoderm) compared to non-deleted (-Cre) *Med28* ^*fl/fl*^ iPSCs colonies (n = 4). Data are presented as mean +/- SD (*p<0.05).

To investigate the role of Med28 in the maintenance of pluripotency, we infected the *Med28* ^*fl/fl*^ iPSCs with Ad-Cre for 24h and plated single cells on gelatin-coated plates (Fig 6A). Colonies formed within 5–8 days were analyzed for Cre-mediated deletion of *Med28* exons 1 and 2 by genotyping using PCR primers described above (Fig 1A). We verified *Med28* deletion after Ad-Cre infection, but observed only heterozygous (*Med28* ^*fl/-*^) or WT (*Med28* ^*fl/fl*^) genotypes among 80 clones tested. An example of genotyping is shown in Fig 6B. We obtained no clones with homozygous deletion of *Med28*, which suggests that iPSCs with homozygous deletion of *Med28* fail to survive, and this is consistent with lack of reprogramming in Med28-deleted MEFs (Fig 4D). More interestingly, heterozygous clones with one copy of *Med28* deleted consistently exhibited differentiated cell morphology, whereas wildtype *Med28* iPSC clones revealed an undifferentiated morphology (Fig 6C). Further, we analyzed *Med28* transcript expression by real-time PCR. As expected, we observed ~50% reduction in *Med28* expression in heterozygous cells (*Med28* ^*fl/fl*^, +Cre) compared to control cells (*Med28* ^*fl/fl*^, -Cre) (Fig 6D). There was a significant decrease in expression of Oct4 and Nanog, both at the transcript and protein levels in colonies where one allele of *Med28* was deleted (Fig 6D and 6E). Taken together, these results conclusively show that unlike *Med28* ^+/-^ embryos, Cre-mediated heterozygous removal of *Med28* in iPSCs leads to loss of their induced pluripotency.

Finally, we analyzed *Med28* heterozygous iPSCs for their ability to differentiate into derivatives of the germ layers using representative makers for three germ layers as well as trophoectoderm by real-time PCR. Transcripts encoding early ectoderm marker *Nestin* and mesoderm marker *T* (Brachyury) were significantly reduced while the primitive endoderm marker *Gata4* was increased after removal of one *Med28* allele (Fig 6F). However, *Med28* ^*fl/fl*^ (+Cre) colonies showed increased expression of *Cdx2* and *Hand1*, suggesting that a decrease in *Med28* level leads to differentiation of iPSCs into the extraembryonic lineage (Fig 6F). Together, these results strongly suggests that Med28 is essential for the self-renewal of iPSCs; while complete removal of Med28 results in loss of survival of iPSCs, acute heterozygous loss of Med28 imposes an extraembryonic trophoectoderm and primitive endoderm fate on the previously pluripotent cells.

## Discussion

As an essential coordinator of Pol II-mediated transcription regulation, the Mediator complex is responsible for integrating signaling events to tissue-specific and cell-specific gene regulation and plays an essential role during development and as well as in disease processes [1, 22, 23]. For example, mutations in MED12 and MED23 in humans cause intellectual disabilities [24, 25], and MED13L mutations cause congenital heart defects [26]. Mediator subunits also play a role in tumorigenesis, as shown for CDK8, which acts as an oncogene regulating β-catenin activity in colorectal cancer [27], MED12, where mutations occur in uterine leiomyomas and prostate cancer [28, 29], and MED29, which has been implicated as an oncogene as well as tumor suppressor in pancreatic cancer [30]. Mouse models with disruption of individual Mediator subunits including Med1, Med12, Med21, Med23, Med24, Med31 and CDK8 are embryonic lethal with distinct developmental defects [23]. In particular, *Med21*- and *Cdk8*-null mice die at very early embryonic stages. *Med21* is essential for embryonic stem (ES) cell survival and disruption results in early peri-implantation embryonic lethality [31], and *Cdk8* knockout mice die at pre-implantation stage E2.5- E3.0 due to fragmented blastomeres and an inability to proceed to compaction [32]. Our work describes for the first time the importance of Med28 in early embryonic development, as well as the role of Med28 in pluripotency. Taken together, it is apparent that KO mice for individual Mediator subunits die at various developmental stages of the embryo with distinct phenotypes, supporting a specific role for each subunit in development [23].

Early embryonic lethality and developmental defects seen in mouse models that lack distinct Mediator subunits are consistent with Mediator being essential for maintenance of ES cell state [9]. Furthermore, recent investigation of enhancers bound by the master transcription factors and Mediator in ES cells revealed that much of enhancer-associated Mediator occupies exceptionally large enhancer domains called “super-enhancers” that were found to contain high levels of ES cell transcription factors Oct4, Sox2, Nanog, Klf4 and Esrrb. These super-enhancers appear to stimulate higher transcriptional activity than typical enhancers and are very sensitive to reduced levels of Med12 [33]. Another recent elegant study that employed circular chromosome conformation capture sequencing to examine a genome-wide, pluripotency-specific interaction network around the Nanog promoter in ESCs, iPSCs and MEFs reported that a large fraction of Nanog-interacting loci was bound by members of the Mediator (Med1 and Med12) and/or cohesin family members in pluripotent cells including iPSCs [34]. Collectively, these studies establish that members of the Mediator complex, in collaboration with cohesin and pluripotency transcription factors, play crucial role in ES cell maintenance and iPSC formation.

Employing reprogrammable MEFs derived from Med28 conditional allele (*Med28fl/fl*), where *Med28* was removed by addition of Cre, we show that Med28 is required for acquisition of iPSCs. We further demonstrate that overexpression of Med28 along with OKSM enhances the reprogramming efficiency of iPSC. Cre-mediated deletion of *Med28* from iPSCs reveals that Med28 is required for the self-renewal. Interestingly, we observe that even heterozygous loss of *Med28* in iPSCs can cause differentiation, which differs from the *Med28* heterozygous knockout mice that are viable, fertile and do not show any obvious phenotype. These results imply that compensatory mechanisms may exist *in vivo* in heterozygous knockout mice, which are lacking in iPSCs where *Med28* is removed in an acute manner with Cre. Further, aberrant differentiation of iPSCs that lack one allele of *Med28* into extraembryonic trophoectoderm and endoderm lineages suggest that Med28 plays a dosage-sensitive role in iPSCs. Based on these observations, we conclude that Med28 will be essential for ES cell viability and self-renewal.

Of final note, we originally identified MED28 as a binding partner of the NF2 protein merlin [3]. Among the Mediator subunits, MED28 is distinct in having a cytosolic role as an NF2 interacting protein as well as a nuclear role as a Mediator complex subunit. Intriguingly, a very recent study has reported a role for Nf2 upstream of Hippo signaling in segregating the ICM from the TE during mouse pre-implantation development [35]. Nf2 is required for the activation of the Hippo pathway leading to Lats1/2-dependent Yap phosphorylation in internal cells of the embryo. Furthermore, in maternal-zygotic *Nf2* mutants, the ICM fails to develop resulting in peri-implantation lethality demonstrating that Hippo pathway activation is absolutely required for the formation of a fully functional ICM [35]. Future studies are necessary to determine whether Med28, in addition to its role as a Mediator subunit in the nucleus, may influence ES cell state and iPSC formation through Nf2/Hippo signaling pathway.

## Materials and Methods

### Targeting vector construction

The targeting vector targeting vector pHHO (a gift from Dr. Henry Ho at Boston Children’s Hospital) was modified by replacing the neomycin-resistant (neo) cassette with two Frt sites flanked by a neo cassette in the reverse orientation of the existing 3’ loxP site. A 5’ loxP site with adapter (NotI-NdeI-loxP-FseI) was cloned into upstream Frt sites flanking the neo cassette. Three homologous arm fragments were PCR amplified from mouse 129 strain genomic DNA using the Expand Long Template PCR System (Roche) following manufacturer’s instructions with PCR primers for 5’-1 arm: 5’-GCGGCCGCCATCAGGGGACCAGACCTAA–3’ and 5’-CATATGCTCCTTTAGAAAAATGAGTCATCAG–3’; for 5’-2 arm: 5’-GGCCGGCCCGCAGAAAAGACTCGGCTAA–3’ and 5’-GTCGACCCAAACAGAGAGCCAGCAAT–3’; for 3’ arm: 5’-GGCGCGCCTTCACTAGCTGACATGTTTTCTGAG–3’ and 5’-GACGTCGGCAGGGGATCATAGATTCA–3’. The PCR products were TA cloned (Invitrogen) into the PCR2.1-TOPO vector and sequenced to confirm the correct sequence. The 5’-1 arm, a 2.2 kb fragment consists of sequence upstream of exon 1, was cloned upstream of the 5’ loxP site using NotI/NdeI restriction sites; the 5’-2 arm, a 2.3 kb fragment spanning *Med28* exons 1 and 2, was cloned into the vector between the 5’ loxP site and the Frt-neo-Frt cassette using FseI/SalI restriction sites, and the 3’-arm, a 3.0 kb fragment spanning *Med28* exons 3 and 4, was cloned downstream of the 3’ loxP site using AscI/AatII restriction sites.

### Gene targeting of ES cells and generation of mutant mice

The targeting vector was linearized with NotI and electroporated into 129/J1 embryonic stem cells by the Mouse Gene Manipulation core of the Intellectual and Developmental Disabilities Center (IDDRC, Children’s Hospital, Boston). G418-resistant clones were screened for homologous recombination by PCR analysis, and out of 192 clones, 3 were identified with correct targeting. All 3 clones were confirmed by Southern blot analysis. Based on karyotyping analysis, one clone with 100% normal karyotype was chosen for transient transfection with Flp recombinase to remove the Frt-neo-Frt cassette. After removal of the Frt-neo-Frt cassette, duplicate plating was used and corresponding non-surviving clones from G418 selection were expanded and genotyped for the conditional allele by PCR and Southern blot analyses. Correctly targeted ES cells were used for blastocyst injection. Chimeras were mated with C57BL/6 female to generate the *Med28* ^*fl/+*^ mouse line. *Med28* ^*fl/+*^ mice were intercrossed to generate the *Med28* ^*fl/fl*^ conditional allele line, which was maintained as a homozygous stock. *Med28* ^*fl/+*^ females were bred with a Protamine-Cre male to generate double heterozygotes/hemizygotes, and males were used to breed with either *Med28* ^*fl/fl*^ females or C57BL/6 females to generate the *Med28* ^*+/-*^ heterozygous knockout mouse line since Protamine-Cre expression is specifically restricted to male germ cells. Finally, *Med28* ^*fl/-*^ and *Med28* ^*+/-*^ mice were intercrossed to generate *Med28* ^*-/-*^ embryos used for experiments. Experimental research protocols were approved by the Institutional Animal Care and Use Committee (IACUC) for the Massachusetts General Hospital (MGH) following the guidelines of the National Institutes of Health for the Care and Use of Laboratory Animals (Protocol # 2004N000314).

### Genotyping by PCR and Southern analysis

DNA was extracted from ES cells using the genomic DNA extraction Gentra kit (Qiagen) following manufacturer’s instructions. Screening of ES cells was performed by PCR analysis using a forward primer outside the targeted region, upstream of the 5’-1 arm: 5’-AACCAAGTCAGAGGGGAGAA–3’ and a targeting vector-specific reverse primer: 5’-TGCTGTCTTGAAAAATCAAAGG–3’. Out of 192 clones screened, 4 clones showed the 5.6 kb expected PCR product confirming correct targeting. The presence of the 5’ loxP site was confirmed in 3 of the 4 positive clones by the presence of a 324 bp PCR product in addition to the 290 bp wildtype product using flanking primers 5’-TCTCAAGCCCTTAATCACCCTA–3’ (primer 1 in Fig 1), and 5’-GTCACATGACGGGCAGTTC–3’ (primer 2 in Fig 1). Genomic DNA from ear punch tissue was used for genotyping three week old pups. An additional PCR analysis confirmed the presence of the mutant allele using primer 1 (above) and 5’-TGCTGTCTTGAAAAATCAAAGG–3’ (primer 3 in Fig 1), which showed a mutant-specific PCR product of 310 bp.

Southern analysis was used to confirm homologous recombination with a 5’ probe (see Fig 1) and a 3’ probe (outside targeting region) following either BspHI or SphI digestion. The presence of the 5’loxP site was also confirmed by Southern blot analysis using 5’ probe following SphI and FseI double digestion. Mouse genomic DNA was digested with either BspHI or SphI and then separated on a 0.9% agarose gel. The DNA was denatured in 0.25M HCl at room temperature for 30 min and transferred overnight to Immobilon-Ny+ transfer membranes (Millipore) in alkaline transfer buffer. Membranes were rinsed for 15 min in 2× SSC, dried on filter paper and UV-crosslinked. Blots were washed in 0.2× SSC and 0.2% SDS at 65°C for 1 h and pre-blocked in hybridization solution (50% formamide, 1× Denhardt solution, 0.5% SDS, 7.5% dextran sulfate, 5× SSC with 80 μg/ml salmon sperm DNA) for 24 h at 65°C. Then membranes were hybridized with the ^32^P-dCTP-labeled DNA probe. The following day, membranes were washed in 2× SSC/0.1% SDS, 1× SSC/0.1% SDS and 0.5× SSC/0.1% SDS each for 1 h at 65°C. Membranes were exposed to X-ray film overnight at -70°C.

### Cell and embryo culture

Mouse ES cells were cultured on gelatin coated tissue culture dishes in ES cell culture media (Dulbecco’s modified Eagle’s medium (high glucose, no sodium pyruvate) supplemented with 15% fetal bovine serum (FBS), 2 mM L-glutamine, 0.1 mM MEM non-essential amino acids, 1mM sodium pyruvate, 0.1 mM 2-mercaptoethanol, 1000 U/mL LIF, containing 100 U/m penicillin and 100 μg/mL streptomycin). The medium was changed every 2 days. Blastocysts were collected by flushing oviducts with Dulbecco’s modified PBS followed by culturing for 3 days on gelatin-coated 96-well plates (for DNA/RNA extraction) or gelatin-coated cover slips (for immunofluorescence) in the same medium as described above. The medium was changed every day with phase contrast microscopic images taken before each medium change. *Med28* ^*fl/fl*^ MEFs were generated following standard protocol [36]. MEFs were cultured in Dulbecco’s modified Eagle’s medium supplemented with 10% FBS containing antibiotics.

### Adenoviral infection of *Med28* ^*fl/fl*^ MEFs

*Med28* ^*fl/fl*^ MEFs were infected with an adenovirus vector expressing the Cre transgene (Ad-Cre) or empty vector as control (Ad-empty) (Univ. Iowa Gene Transfer Vector Core). Briefly, cells were serially infected two sequential days at MOI250 in serum free DMEM for 6–8 hours, followed by removal of virus and media change to complete medium (DMEM with 10%FBS). At post-infection day 4 (96 h after first infection), cells were trypsinized and plated in order to carry out subsequent experiments.

### Immunofluorescence analysis

For immunofluorescence (IF) analysis of *Med28* ^*fl/fl*^ MEFs, cells were first infected using Ad-empty or Ad-Cre (see methods above). For staining of blastocyst outgrowths, blastocysts were recovered, plated and allowed to grow for 3 days *in vitro* (DIV3) prior to IF staining as described previously [16] with minor modifications. All IF procedures were carried out at 37°C unless otherwise indicated. Adenovirus-infected MEFs and attached DIV3 blastocysts were fixed in 4% paraformaldehyde (PFA)/phosphate-buffered saline (PBS) for 30 min, permeabilized in 0.25% Triton X–100/PBS for 20 min at room temperature and blocked in 10% normal goat serum (NGS)/PBS for 45 min. MEFs were incubated with a primary antibody against Med28 (7E1; 1:100) [3]. Blastocysts were incubated with primary antibodies against Med28, Oct–4 (Cell Signaling Technology; 1:400), Nanog (Abcam;1:100) and GATA4 (Santa Cruz Biotechnology; 1:100) in 1% BSA/PBS. After several washes with PBS, incubation with secondary antibodies (Alexa Fluor 594 goat anti-mouse, Alexa Fluor 488 goat anti-rabbit, or Alexa Fluor 594 goat anti-rabbit; Molecular Probes, Invitrogen) was performed for 30 min according to manufacturers suggestions. Coverslips were mounted on slides using the ProLong Gold Antifade with DAPI (Molecular Probes, Invitrogen). Images were obtained using a Nikon Inverted microscope (Eclipse TE2000-U) and NIS-Elements BR 3.2 imaging software for Oct4 and Nanog staining, and a Leica TCS SP5 AOBS scanning laser confocal microscope (Leica Microsystems) for GATA4 staining.

### MEF reprogramming and AP staining

Lentiviruses for STEMCCA, rTTA, Med28, and GFP were packaged using Fugene 6 (Promega) following manufacturer’s instructions. *Med28* ^*fl/fl*^ MEFs (1x10^5^ cells P2) were seeded on gelatin-coated 6-well plates and cultured overnight. STEMCCA, rTTA with GFP or Med28 were co-infected the second day. On day 3, 1X10^4^ cells were seeded to each well of a 6-well plate with ES cell culture media supplemented with Doxycycline and vitamin C. Medium was changed every 2 days. For deletion of *Med28* (exons 1 and 2), one day after Dox treatment, cells were infected with Ad-Cre or Ad-empty as described above. After Dox treatment for 12 days, cells were cultured in ES cell culture medium for an additional 5 days. Single colonies were picked and cultured on MEF feeders in ES cell culture medium. Reprogramming of OKSM-MEFs was performed as above except using a single infection with either GFP or Med28. 6 days after removal of Dox, cells were fixed with 4% paraformaldehyde, and alkaline phosphatase (AP)-staining was performed following manufacturer’s instructions (Millipore).

### RT-PCR and real-time PCR

RNA was extracted from either 3.5 dpc (E3.5) blastocysts or cultured DIV3 blastocysts using the Absolutely RNA Nanoprep kit (Agilent) following manufacturer’s instructions. RNA from MEFs and iPS cells was extracted using the RNeasy kit (Qiagen) following manufacturer’s instructions. cDNA was synthesized using Superscript III (Invitrogen) following the manufacturer’s instructions. Real-time PCR was performed using the Cycler 480 (Roche) with 2X Syber Green master mix (Roche) following manufacturer’s instructions. Due to a limited amount of RNA obtained from intact and cultured blastocyst, real-time PCR was first performed for *Gapdh*, and then semi-quantitative RT-PCR analysis was performed using samples diluted to the same concentration based on *Gapdh* quantification

### Western blot analysis

Antibodies used included our previously reported 7E1 anti-Med28 mouse monoclonal [3], and Gapdh (EMD Millipore). At post-infection day 6 *Med28* ^*fl/fl*^ MEFs infected with Ad-empty or Ad-Cre were lysed in 1% NP40 lysis buffer (1% NP40, 150mM NaCl, 50mM Tris-HCl, pH8.0), proteins were separated by 4–15% SDS-Page (Bio-Rad) and transferred to nitrocellulose membrane (Bio-Rad) for immunoblotting. All lysis buffers contained 1x HALT^TM^ phosphatase inhibitor cocktail (Thermo Scientific) and 1x Complete^TM^ protease inhibitor cocktail (Roche).

### Histological analysis

Uteri from pregnant mice were fixed in Bouin’s fixative overnight, dehydrated, and embedded in paraffin by the Dana Farber/Harvard Cancer Center Rodent Histopathological Core. Sections (5 μm) were stained with hematoxylin and eosin by standard procedures.

In situ *hybridization of whole embryos—*Briefly, E8.0–11.5 embryos of mouse strain CD1 were dissected, fixed in 4% PFA/PBS and processed for whole mount *in situ* hybridization as described [37]. The *Med28* in situ hybridization probe spans a 679 bp fragment (bp115-793 of the mouse *Med28* mRNA, NM_025895). Pictures were taken with an Olympus SZX12 binocular stereomicroscope mounted with CCD cameras.

### Statistical analysis

All experiments were performed in triplicate. Data values are represented as mean +/- SD. Within each group, student t-test was performed with a value of *p*<0.05 considered significant.

## Supporting Information

S1 Fig *Med28* expression pattern during early mouse embryonic development. Whole mount *in situ* hybridization analysis shows broad tissue expression of *Med28* during early mouse embryonic development including CNS (fore-, mid-, hindbrain), spinal cord, dorsal root ganglion, muscle precursors in the limbs, somites and heart. drg: dorsal root ganglia; hf: head fold; ht: heart; sc: spinal cord; som: somites; sv: sinus venosus. The arrow points to the inflow tract of the heart.
(TIF)

S2 Fig Med28 removal or overexpression does not change cell number during reprogramming. **(A)** *Med28* ^*fl/fl*^ MEFs were infected with OKSM and rtTA, plated at 1x10^5^ cells on 35mm plates one day after infection, and then treated with Dox for 1 day. Cells were then infected with either Ad-empty or Ad-CRE (to removed Med28) and cultured for an additional 5 days. **(B)** *Med28* ^*fl/fl*^ MEFs were infected with OKSM, rtTA and GFP (OKSM+GFP) or OKSM, rtTA and Med28 (OKSM+Med28), plated at 1x10^5^ cells on 35mm plates one day after infection, and then treated with Dox for 5 days. No observable difference in cell number was found for Med28-deficient (A, Ad-Cre) or Med28-overexpression (B, OKSM+Med28) cells compared to respective controls. Cell number quantitation is shown, and data are presented as mean +/- STDEV.
(TIF)
