# Supplementary figures and images for: Mediator Subunit Med28 Is Essential for Mouse Peri-Implantation Development and Pluripotency
Source: PLoS One. 2015 Oct 7;10(10):e0140192. doi: 10.1371/journal.pone.0140192 (PMC4596692; doi:10.1371/journal.pone.0140192)

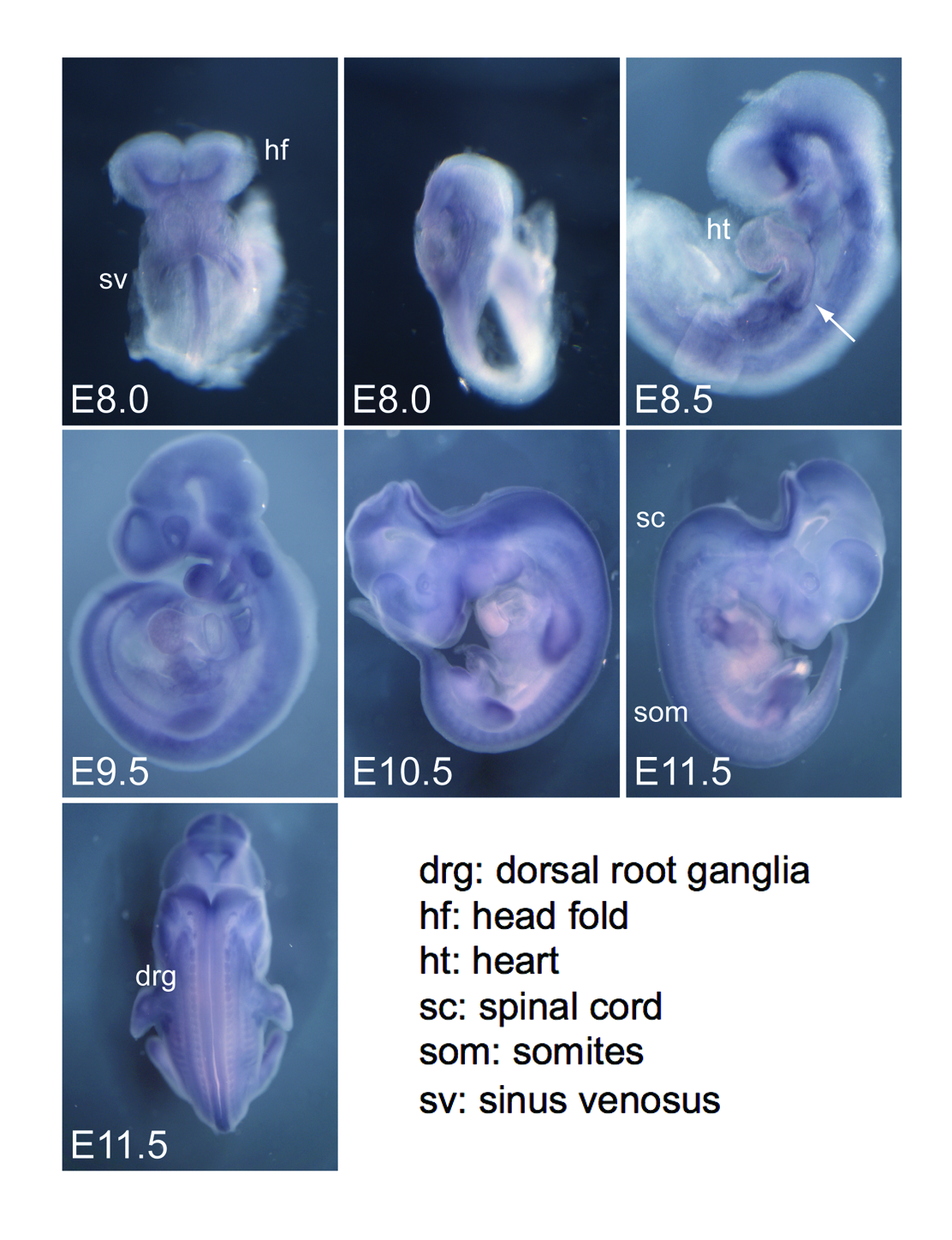

Supplement: S1 Fig — Whole mount in situ hybridization analysis shows broad tissue expression of Med28 during early mouse embryonic development including CNS (fore-, mid-, hindbrain), spinal cord, dorsal root ganglion, muscle precursors in the limbs, somites and heart. drg: dorsal root ganglia; hf: head fold; ht: heart; sc: spinal cord; som: somites; sv: sinus venosus. The arrow points to the inflow tract of the heart. (TIF) [file pone.0140192.s001.tif]

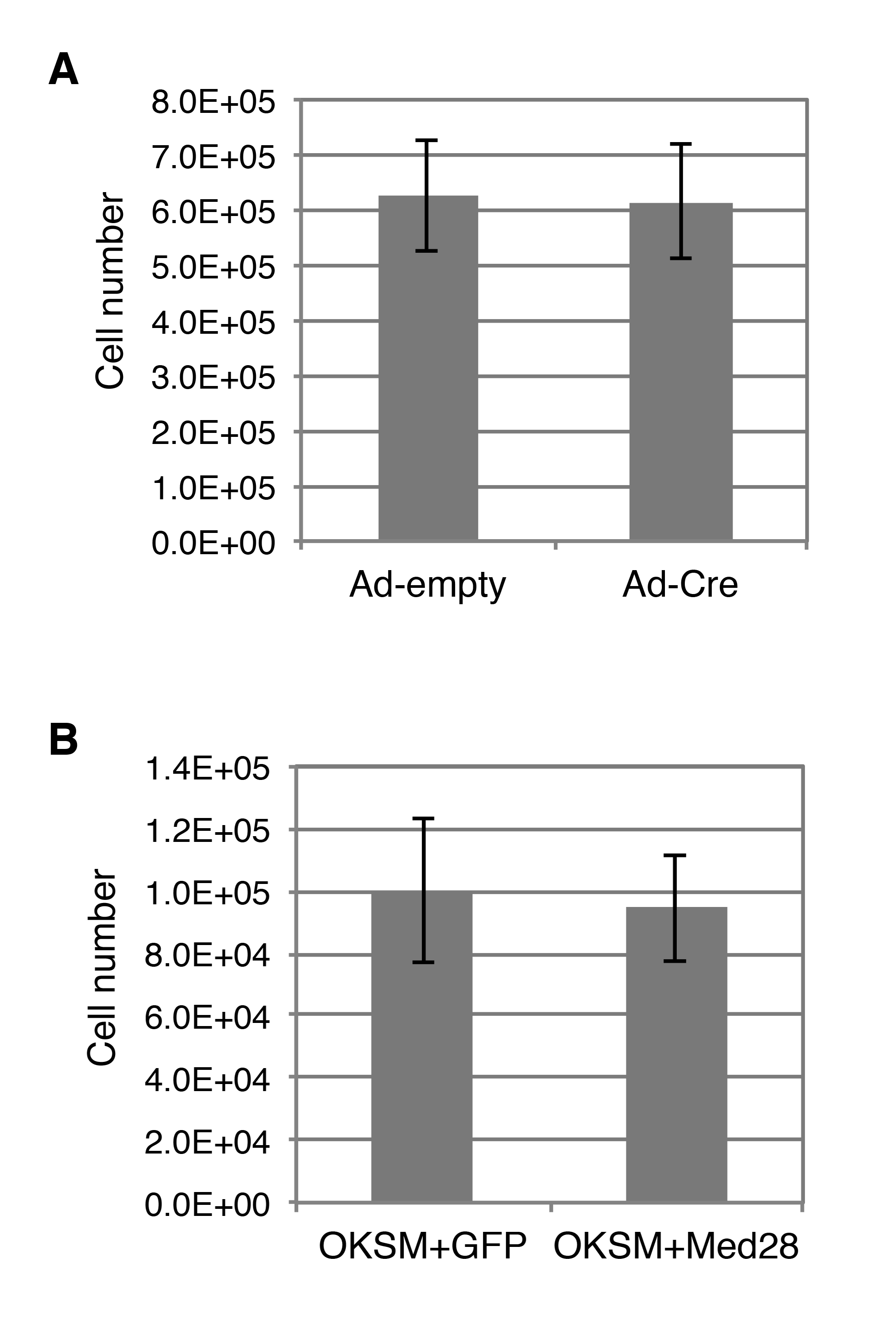

Supplement: S2 Fig — (A) Med28 fl/fl MEFs were infected with OKSM and rtTA, plated at 1x10^5^ cells on 35mm plates one day after infection, and then treated with Dox for 1 day. Cells were then infected with either Ad-empty or Ad-CRE (to removed Med28) and cultured for an additional 5 days. (B) Med28 fl/fl MEFs were infected with OKSM, rtTA and GFP (OKSM+GFP) or OKSM, rtTA and Med28 (OKSM+Med28), plated at 1x10^5^ cells on 35mm plates one day after infection, and then treated with Dox for 5 days. No observable difference in cell number was found for Med28-deficient (A, Ad-Cre) or Med28-overexpression (B, OKSM+Med28) cells compared to respective controls. Cell number quantitation is shown, and data are presented as mean +/- STDEV. (TIF) [file pone.0140192.s002.tif]
